# Skin Color, Cultural Capital, and Beauty Products: An Investigation of the Use of Skin Fairness Products in Mumbai, India

**DOI:** 10.3389/fpubh.2017.00365

**Published:** 2018-01-23

**Authors:** Hemal Shroff, Phillippa C. Diedrichs, Nadia Craddock

**Affiliations:** ^1^Centre for Health and Social Sciences, School of Health Systems Studies, Tata Institute of Social Sciences, Mumbai, India; ^2^Centre for Appearance Research, University of the West of England, Bristol, United Kingdom

**Keywords:** skin lightening, side effects, media pressure, skin color, skin-whitening products, cosmetics

## Abstract

The use of skin fairness products that frequently contain toxic ingredients is associated with significant adverse health side effects. Due to the high prevalence of use in Asian and African countries, skin fairness product use is recognized as a growing public health concern. The multi-million-dollar skin fairness product industry has also been criticized for perpetuating racism and social inequalities by reinforcing beliefs about the benefits of skin fairness for cultural capital. No quantitative studies have assessed people’s beliefs about fairness and reasons for using or not using these products in India, one of the largest global markets for skin fairness products. The current study explored skin fairness product use among 1,992 women and men aged 16–60 years in the city of Mumbai, India using a self-report questionnaire. A total of 37.6% of the sample reported currently using skin fairness products, with women being two times more likely to use these products. Among current users, 17% reported past experiences of adverse side effects, and “Media/TV/Adverts” were the most common prompts for using fairness products, followed by “Friends” and “Family.” Men were significantly more likely than women to endorse beliefs about fairness being more attractive and were more likely to perceive family and peers as viewing fairness as beneficial for cultural capital. There were no differences between women and men currently using products in their desire to look as fair as media celebrities. Among non-users, women were significantly more likely than men to report concerns about product efficacy and side effects as reasons for non-use, while men were significantly more likely to report socioeconomic reasons for non-use. Implications of these findings are discussed in light of growing public health concerns about the use of fairness products, and potential for advocacy and public health interventions to address the use of skin fairness products.

## Introduction

Studies have documented the use of skin fairness products, sometimes referred to as “skin whitening products,” “skin bleaching products,” “depigmenting agents,” in Africa, Europe, North America, and Asia, with prevalence of use ranging from 27 to 77% among community samples (1). Skin fairness products include whitening and skin-lightening creams, face washes, deodorants, and lotions. This industry is one of the fastest growing segments of the global beauty industry, particularly in Asia and Africa, with marketing forecasters predicting it will be worth an estimated $US 31.2 billion by 2024 (2). Historically marketed to women, companies have recently expanded their offerings to include products designed and marketed specifically for men. Advertisements and packaging overtly claim that products will make consumers’ skin fairer and more even-toned, while product names and the use of well-known models and actors in advertisements imply that they will enhance consumers’ cultural capital *via* improvements in attractiveness, youthfulness, confidence, and success (3, 4). Cultural capital refers to social and cultural assets (e.g., education, style of speech and dress, intellect, and appearance) that can enhance an individual’s social mobility in stratified societies (5).

The widespread use of skin fairness products is increasingly recognized as a public health, environmental justice, and social justice issue due to the deleterious health side effects and the potential reinforcement of racial and social inequalities (6, 7). Despite this, empirical research into skin fairness product use is limited to prevalence and medical side effect investigations, especially among samples in Asia (1). This is particularly the case for research conducted in India, one of the fastest growing markets with an annual spend of $US 450 million on skin fairness products (8). Recognizing the social and public health implications of fairness product use, this study presents an examination of women’s and men’s use of skin fairness products, and their beliefs about fairness in a metropolitan area in India.

The relevance of skin fairness products to public health is highlighted by the scope of the industry, the widespread use of these products, and the potential health risks associated with their use. The type and extent of side effects depends on the nature and concentration of product ingredients (9). While some cosmetic products are associated with lower risk, other products contain highly active and potentially dangerous ingredients, such as hydroquinone, mercury, and bleaching agents such as hydrogen peroxide (10, 11). Agarwal et al. (12) tested 23 skin fairness face creams available in India and found that almost 50% of these creams (*n* = 11) contained steroids that can be harmful to skin. A subsequent study found that levels of mercury in many popular face creams are increasing over time (13).

Side effects of skin fairness products containing hydroquinone, steroids, or mercury can include irritation, inflammation, thinning of skin, scarring, abnormalities among newborn babies if used during pregnancy and breast-feeding, and kidney, liver, or nerve damage (14, 15). Skin-bleaching agents also increase susceptibility to infections including bacteria, fungus, parasites, and viruses (16). Some countries (e.g., Ghana, Ivory Coast, Nigeria, South Africa, and Zimbabwe) have banned the import of fairness products that contain hydroquinone and mercury (17). Nevertheless, many countries, including the two biggest markets, India and China, do not have regulations on ingredients contained within these products. The widespread use of skin fairness products presents a growing public health concern, particularly in Asia.

In several Asian countries and cultures, white or fair skin is perceived to be more attractive and desirable due to its social advantages for marital and career prospects (1, 18). This is particularly relevant in Asian countries, including India, Japan, Korea, China, and Thailand, where skin fairness has been understood to be a cultural marker of class, wealth, and social status for centuries (1). The advertising industry in particular, is argued to play a significant role in reinforcing and capitalizing on stereotypical notions of caste, age, race, and beauty. Shankar et al. (19) assert that the advertising of fairness products is akin to “disease mongering,” not necessarily creating a market where there is not one, but playing on people’s insecurities about appearance and making huge profits from them. A content analysis of advertisements for skin-related products in women’s magazines in India, Japan, Korea, and Hong Kong found that “good skin” was depicted as “*smooth, young, pore-less, line-free, bright, transparent, white, full and fine”* and advert narratives suggested enhanced cultural capital through the use of products advertised to achieve fairer skin (4). Thus, fairer skin may be assumed to improve career and marital prospects and increase cultural capital in societies that value fair skin.

India presents a particularly interesting case example due to its large market share in the skin fairness industry and its ancient cultural notions of beauty and fairness, which have strong ties with caste and community biases, whereby fairer skin is preferred (20). In India, the market was liberalized toward the end of the twentieth century, which led to a surge in the availability of fairness products. At present, there are many fairness creams, face washes, and lotions for men and women widely available, including products marketed by local and international brands. The skin fairness industry currently represents 50% of India’s entire skincare market, with estimates of its worth varying between $US 450–535 million (8, 21).

Indian consumers are led to believe that fairer skin will provide them with higher status, and advertisements serve to reinforce this notion (21, 22). Phillips (23) describes the societal equation of fairness with beauty and the accompanying negative connotation of lack of beauty associated with darker skin color in India. She also discussed the associated moral and behavioral qualities linked with skin color, highlighting the far-reaching impact fair skin may have on an individual’s life and cultural capital within the Indian context. Individuals with darker skin in India are often assumed to be blue-collar or manual workers, required to spend time outdoors where their skin becomes darker. Furthermore, darker skin color has been associated with adverse moral and behavioral qualities (4, 23). These factors are compounded by two centuries of colonial “white” rule (20). Consequently, there are strong ties between caste, economic class, marital prospects, occupation status, colonialism, and skin color in India.

Although theoretical critiques have examined the marketing and use of skin fairness products in India, empirical quantitative research on skin fairness product use, and the social and psychological factors driving this is sparse. Most quantitative studies [e.g., Ref. (24)] investigating the prevalence of, and reasons for, using skin fairness products have been conducted in Sub-Saharan Africa (1). Nonetheless, a few studies indicate that use of these products is prevalent and associated with social disadvantage and poor health correlates among Indian consumers. Indian women and men were included in a 26-country study that investigated the prevalence and correlates of using skin fairness products among undergraduate students (16). In their study, the authors found that 18.9% of 799 Indian undergraduate students sampled reported using skin fairness products, and their use was associated with depression, risky sexual behaviors, lack of personal control, and low social support. Furthermore, a recent experimental online study found that women in India who were primed temporarily to feel disempowered were likely to indicate a stronger preference for medically risky skin fairness products (i.e., those containing more active and potentially harmful ingredients) as compared to less risky cosmetic products, in addition to finding the products more relevant and useful (25).

To inform future research, advocacy, and public health intervention efforts, the current study was undertaken to explore the use and non-use of skin fairness products in a large, educated, community sample of women and men in Mumbai, India. Among self-identified current users and non-users, reasons for use or non-use were assessed and beliefs about the benefits of skin fairness were also explored. Gender differences in reasons for and against using fairness products were also explored. Given that beauty products have historically been marketed to women and that women tend to be more likely to be judged on the basis of their appearance, experience worse body image, and have less social capital than men (4, 7), it was hypothesized that women would be more likely to report use of skin fairness products than men and would be more likely to endorse the importance of skin fairness for attractiveness and cultural capital than men.

## Materials and Methods

### Participants

The sample consisted of 1,992 adults (62% women) from Mumbai, India. The mean age of the entire sample was 24.69 years (SD = 9.06; range 16–60 years). The distribution of occupations was such that 1,217 (61%) were students, 172 (8.6%) were professionals, 257 (12.9%) were employed (other than professionals), 225 (11.3%) were homemakers, 65 (3.3%) were involved in business, and the rest (2.9%) did not provide data on their occupation. There were 748 participants (542 women, 206 men, and 6 did not report gender) who reported that they had used skin fairness products in the past 30 days (current users). The mean age among current users was 24.73 (SD = 9.12). The remaining 1,238 participants (696 women, 540 men, and 2 had not specified gender) had not used fairness products in the past 30 days (non-users). The mean age of non-users was 24.67 years (SD = 9.03). See Table 1 for more demographic information.

**Table 1:** Demographic information.

	Total sample	Current users	Non-users
	1,992	754 (37.9%)	1,238 (62.1%)
**Gender**			
Men	746 (37.7%)	206 (27.3%)	540 (43.6%)
Women	1,238 (61.9%)	542 (71.9%)	696 (56.2%)
Missing	8 (0.4%)	6 (0.8%)	2 (0.2%)
**Age range**			
16–17	133 (6.7%)	56 (7.4%)	77 (6.2%)
18–24	1,263 (63.4%)	475 (63.0%)	788 (63.7%)
25–34	324 (16.4%)	124 (16.0%)	200 (16.2%)
35–44	137 (6.9%)	42 (5.4%)	95 (7.7%)
45–54	112 (5.6%)	49 (5.6%)	63 (5.1%)
55–60	23 (1.3%)	8 (1.1%)	15 (2.4)
**Mean age (SD)**			
Total	24.69 (9.06)	24.73 (9.12)	24.67 (9.03)
Men	23.92 (8.36)	23.70 (8.57)	24.01 (8.29)
Women	25.19 (9.45)	25.18 (9.31)	25.20 (9.55)
**Occupation**			
Student	1,217 (61.0%)	437 (58.0%)	780 (63.0%)
Professional	172 (8.6%)	65 (8.6%)	107 (8.6%)
Employed (other than professional)	257 (12.9%)	117 (15.5%)	140 (11.3%)
Homemaker	225 (11.3%)	102 (13.5%)	123 (9.9%)
Business	65 (3.3%)	24 (3.2%)	41 (3.3%)
Missing	56 (2.9%)	9 (1.2%)	47 (3.9)

### Measures

Participants completed a self-report questionnaire in their home, classroom, or workplace.

#### Demographics

Participants self-reported their gender, age, and occupation within the questionnaire.

#### Use of Skin Fairness Products

Participants were asked if they had ever used skin fairness products in their lifetime (“*How often in your lifetime have you used fairness products?*”). The response format was 0 = “*Not at all*,” 1 = “*Less than one month*,” 2 = “*1*–*3 months*,” 3 = “*4*–*6 months*,” 4 = “*more than six months*.” They were also asked about their frequency of use in the last 30 days (“*On how many occasions in the past thirty days have you used fairness products?*”). The response format for this question was 0 = “*Not at all*,” 1 = “*1*–*2 times a week*,” 2 = “*3*–*4 times a week*,” 3 = “*Everyday*,” 4 = “*More than once a day*.” For subsequent analyses, non-users were defined as those who reported that they had used fairness products “*Not at all”* in the last 30 days. Current users were defined as those who reported any use of fairness products in the past 30 days.

For current users, further items asked the respondents about who had introduced them to fairness products (open-ended response format), the age at which they first started using fairness products, and their main reason for using fairness products (open-ended response format). They were also asked if they had experienced any adverse side effects after using fairness creams and whether they had consulted a health professional either before or after use (“*Yes*” or “*No*” response formats).

#### Beliefs about Fairness

Beliefs about fairness were assessed with a purpose built scale, as there was no widely used standardized scale available. The first author (Hemal Shroff) created the scale with feedback from doctors, academics, and statistical consultants. Items were pilot tested with a sample of 10 individuals to ensure comprehension of the items. No changes were necessary based on the pilot testing. Computation of means and SDs was done for the demographic items, frequency of use, and the user and non-user scales.

Participants who reported current (within the past 30 days) use of fairness creams were asked to complete a 15-item scale (The Usage of Fairness Products Scale; see Table 2). Three subscales assessed beliefs in relation to “body image and attractiveness” (e.g., “*The fairer I am, the more attractive I feel*”), “family and peer influence” (e.g., “*I try to look fair because my family members say it is attractive*”), and “media and celebrity influence” (e.g., “*I wish I was as fair as the people (actors) on TV and in ads*”). Subscales were developed conceptually on the basis of prior body image research and theory [e.g., Ref. (26)]. Principal components analysis revealed three factors congruent with these subscales, with satisfactory factor loadings (*item coefficients* <0.50) for statements onto their respective subscales. Participants were asked to rate on a Likert scale the extent to which they agreed with the statements (1 = strongly disagree; 5 = strongly agree) contained within each subscale. Mean total subscale scores were calculated with higher scores indicating greater endorsement of the beliefs about fairness. Internal consistencies for the subscales were good among women and men (Cronbach’s α = 0.77–92). Mean and SD scores for items for the male and female users on each subscale are included in Table 2.

**Table 2:** The Usage of Fairness Products Scale.

Subscales	Total sample	Women	Men
	Mean (SD)	Mean (SD)	Mean (SD)
**Body image and attractiveness subscale**			
I use fairness products because they make me more attractive			
I use fairness products because they make me more confident about my appearance			
The fairer I am, the more attractive I feel			
I look thinner when I am fairer			
I will have better career prospects if I am fairer			
*Subscale Total Mean Score*	2.97 (0.99)	2.90 (0.98)	3.12 (1.00)

**Family and peer influence subscale**			
I want to look fairer because people in my family think it makes my skin look nice			
My friends say members of the opposite sex will find me more attractive if I am fairer			
My family members say I will be more attractive to a marriage partner if I am fairer			
I would like to be fair because my friends and/or peers say it is attractive			
I try to look fair because my family members say it is attractive			
*Subscale Total Mean Score*	3.09 (1.15)	2.99 (1.16)	3.31 (1.08)

**Media and celebrities subscale**			
I would like my skin tone to be fair like people (actors) in TV, ads and movies			
I wish I was as fair as the people (actors) on TV and in ads			
I try to be fair like famous people I see in magazines			
I want to be as fair as the people in magazines			
I try to be as fair as the people (actors) in movies			
*Subscale Total Mean Score*	2.87 (1.25)	2.82 (1.24)	2.97 (1.25)
Total Scale Score	2.98 (0.95)	2.90 (0.94)	3.14 (0.96)

Participants who reported they had not used fairness products in the last 30 days (i.e., non-users) were asked to complete an 8-item scale (The Non-Usage of Fairness Products Scale; see Table 3). Two subscales assessed beliefs in relation to “product efficacy and side effects” (e.g., “*I don’t use fairness products because they are harmful for my skin”*) and “socio-economic factors” (e.g., “*I don’t use fairness products because I cannot afford them”*). Principal components analysis revealed two factors congruent with these subscales. However, while the factor loading for the first subscale “product efficacy and side effects” was satisfactory (item coefficients <0.55), the results were poor for the “socio-economic factors” subscale (item coefficients <0.35). Participants were asked to rate the extent to which they agreed with the statements within each subscale on a Likert scale (1 = strongly disagree; 5 = strongly agree). Mean subscale total scale scores were calculated with higher scores indicating greater endorsement of reasons for not using fairness creams. Internal consistency values for the “product efficacy and side effects” subscale were satisfactory among women and men (Cronbach’s α = 0.69–0.71), although they were inadequate for the “socio-economic factors subscale” (Cronbach’s α = 0.44–0.48). Mean and SD scores for items for the men and women non-users are included in Table 3.

**Table 3:** The Non-Usage of Fairness Products Scale.

Item	Total sample	Women	Men
	Mean (SD)	Mean (SD)	Mean (SD)
**Product efficacy and side effects subscale**			
I don’t use fairness products because I think they do not work			
I don’t use fairness products because they are harmful for my skin			
I don’t use fairness products because I don’t know what the long-term effect of using these products is			
I don’t use fairness products because once you start you have to keep using them			
I am concerned that my pimples will get worse with the use of fairness products			
*Total Mean Subscale Score*	3.46 (0.90)	3.55 (0.88)	3.36 (0.92)

**Socioeconomic reasons subscale**			
I don’t use fairness products because I do not wish to be fairer			
I don’t use fairness products because I do not like the model/star promoting it			
I don’t use fairness products because I cannot afford them			
*Total Mean Subscale Score*	2.58 (0.99)	2.51 (0.98)	2.65 (1.00)
Total Scale Score	3.13 (0.75)	3.16 (0.72)	3.09 (0.78)

### Procedure

Participants were asked to complete the questionnaire in one setting after obtaining consent, either in their homes, workplaces, or in their classrooms. In accordance with ethical guidelines governing research in India, it was not necessary to obtain approval for the study from an ethical review board, especially as the study did not involve data collection with vulnerable participants and had no funding source. Nevertheless, all the procedures followed in the study complied with the guidelines laid down by ethics bodies in India. The participants were informed that the study was an exploratory investigation of the use of fairness products in India and that they would be asked to complete a questionnaire on their reasons for use or lack of use of fairness products. Participants were given information on the purpose of the study. They were informed that there would be no compensation for taking part in the study and that there were no known risks to participating. They were also informed that participation was voluntary. No identifying information was collected from the participants, as such the questionnaires were anonymous and the risk of data protection issues was minimized. Once verbal consent was obtained, participants were provided with a paper version of the questionnaire and were asked to complete it in the presence of a research assistant. The questionnaires were administered in English. One large educational institution provided the researchers with permission to collect data in their classrooms. Data from the community sample (other than the educational institution) were collected from apartment complexes where the researchers were provided with permission to collect data. Certain companies also provided permission to collect data from their employees.

## Results

### Use of Skin Fairness Products

#### Frequency

Of the total sample (*N* = 1,992), 1,084 (54.4%) participants had used fairness products within their lifetime and 901 (45.2%) had never used fairness products. Lifetime use data was missing for seven participants. There was a significant association between gender and lifetime use of fairness products, $\chi_{\left(1\right)}^{2}=34.15$, *p* < 0.001. Women were 1.7 times more likely to have ever used skin fairness products than men. Specifically, 59.6% of the women had used fairness products at some point in their lifetime, while 46.1% of the men sampled had used fairness products at some point in their lifetime.

Of the total sample, 754 (37.9%) participants had used fairness products within the past 30 days (i.e., were deemed current users) and 1,238 (62.1%) had not (i.e., were deemed non-users). Among current users, 32.7% (*n* = 355) reported using fairness products every day or more than once a day. There was a significant association between gender and current use of fairness products, $\chi_{\left(1\right)}^{2}=51.80$, *p* < 0.001. Women were 2.04 times more likely to be currently using skin fairness products than men. Specifically, 43.8% of the women sampled, currently used fairness products, while 27.6% of the men in the sample currently used fairness products. Women were also significantly more likely to use fairness products on a more frequent basis, $\chi_{\left(3\right)}^{2}=55.20$, *p* < 0.001, with women representing 74.6% (*n* = 265) of daily users.

#### Age

There was no significant difference in age between current users (M = 24.73, SD = 9.12) and non-users (M = 24.67, SD = 9.03), *t*_(1,990)_ = 0.128, *p* = NS. There was also no difference in age between women (M = 25.34, SD = 10.11) and men who were non-users (M = 24.87, SD = 10.40), *t*_(1,263)_ = −0.814, *p* = NS. Among current users, however, the men sampled (M = 23.70, SD = 8.57) were significantly younger than the women sampled (M = 25.18, SD = 9.31), *t*_(400)_ = −2.06, *p* < 0.05. Consequently, age was controlled for in the analyses comparing beliefs about fairness between women and men. Among current users, the mean age of initiating use of fairness products was 18.56 years (SD = 6.08, range = 10–57). There was no significant difference in the age of starting use of fairness products by gender *t*_(713)_ = −1.35, *p* = NS.

#### Side Effects

A substantial minority of current users (17%; *n* = 128) reported adverse side effects after the use of fairness products, with 3.1% having sought help from a health professional. Women were significantly more likely to report having experienced side effects than men, *t*_(279)_ = −2.96, *p* < 0.01.

#### Reasons for Use

When current users were asked “*who or what prompted you to start using fairness products*,” almost half (44.6%) responded “*Media/TV/Advertisements*” prompted their use, while 20.6% reported that “*Friends*,” “*Family*” (16.4%), “*Other/Self”* (9.5%), and “*Health Professionals*” (1.3%) prompted their first use of fairness products (see Table 4). There were no significant differences in the source of prompts among women and men $\chi_{\left(4\right)}^{2}=5.65$, *p* = NS. When current users were asked about their “*main reason for using fairness products*,” desire to be fairer was the most common reason, followed by desire to look more beautiful/attractive, to moisturize/protect skin, social pressures, and other reasons. There was a significant difference in the reported reasons for fairness product use between women and men $\chi_{\left(4\right)}^{2}=11.34$, *p* < 0.05. Women reported using fairness products for beauty/attractiveness reasons more often than men (36.4 vs 26.5%, respectively), while more men reported using fairness products due to social pressures as compared to women (5.1 vs 1.8%, respectively).

**Table 4:** Current users’ self-reported prompts and reasons for using fairness products.

Item	Total sample, *N* (%)	Women, *N* (%)	Men, *N* (%)
**Prompts**			
Media/TV/ads	336 (44.6)	241 (48.4)	91 (47.2)
Family	124 (16.4)	90 (18.1)	34 (17.6)
Friends	155 (20.6)	117 (23.5)	37 (19.2)
Professionals	10 (1.3)	6 (1.2)	3 (1.6)
Other/self	72 (9.5)	44 (8.8)	28 (14.5)
*Missing*	57 (7.6)		
**Reasons**			
To look beautiful/attractive	236 (31.3)	184 (36.4)	52 (26.5)
To look fair	273 (36.2)	191 (37.3)	77 (39.3)
To moisturize/protect skin	92 (12.2)	62 (12.3)	30 (15.3)
Social pressures	19 (2.5)	9 (1.8)	10 (5.1)
Other (e.g., trial)	87 (11.5)	60 (11.9)	27 (13.8)
*Missing*	47 (6.2)		

### Beliefs about Fairness

#### Current Users

To understand the extent to which users endorsed beliefs that being fair is more attractive (“body image and attractiveness” subscale), that their family and friends perceived fairness as desirable and better for cultural capital (“family and peer influence” subscale), and they had “a desire to look fair like media and celebrities” (“media and celebrity influence” subscale), the frequencies with which women and men reported subscale means of 4 or above (i.e., they endorsed “agree” or “strongly agree” for the subscales) were examined (see Table 2 for mean subscale scores). Women and men most strongly endorsed family and peer ideas about fairness being desirable with 26.7 and 33.4%, respectively, either agreeing or strongly agreeing with subscale statements on average. This was followed by a desire to look fair like people shown in the media and celebrities where 25.1 and 28.1% of women and men, respectively, agreed to strongly agreed on average to these statements. Finally, 25.4% of men and 13.2% of women agreed to strongly agreed on average to statements endorsing beliefs that being fair is more attractive.

Analyses of covariance with Bonferroni corrections were conducted to determine if there was a significant difference in the extent to which women and men currently using fairness products endorsed different beliefs. Men were significantly more likely to endorse ideas about fairness being more attractive, *F*_(1, 744)_ = 6.91, *p* < 0.01, and they were significantly more likely to endorse family and peer influences, *F*_(1, 742)_ = 11.75, *p* < 0.01. However, there was no difference between women and men in the extent to which they reported a desire to look as fair as people depicted in the media and celebrities, *F*_(1, 740)_ = 1.72, *p* = NS.

#### Non-Users

Women and men not currently using fairness products most strongly endorsed concerns about product efficacy and side effects as reasons for not using fairness products, followed by socioeconomic factors. To elaborate, 29.6% of men and 39.2% of women on average agreed or strongly agreed with items included in the product efficacy and side effects subscale. Meanwhile, 11.7% of men and 10.3% of women on average agreed or strongly agreed with items included in the socioeconomic reasons subscale. Women were significantly more likely than men to endorse concerns about side effects and product efficacy, *t*_(1,277)_ = −3.785, *p* < 0.001, while men were significantly more likely to endorse socioeconomic reasons for not currently using fairness products than women, *t*_(1,247)_ = 3.24 *p* < 0.01. See Table 3 for the mean subscale scores for women and men for reasons for not using skin fairness products.

## Discussion

This study was conducted to explore the use of fairness products, beliefs about fairness, and reasons for using and not using these products, among a sample of women and men in the city of Mumbai, India. The use of fairness products is of public health concern in Asian countries because of the high prevalence and reported side effects (1, 12), along with the reinforcement of racism and social disparities. This was a convenience sample and there were more women and students. Thus, the results may be somewhat skewed by their over representation. Although there were significantly more women than men using fairness products, a little over a quarter of the men sampled reported current use of fairness products. Thus, the number of male users was substantial. Given that there were no fairness products created specifically for men 20 years ago, these findings suggests that the availability, combined with advertising that reinforces societal stereotypes, may have led to use among a traditionally ignored population for skin fairness products.

More than half of the entire sample reported that they had used fairness products at some point in their lifetime. Women were more likely to have used fairness products in their lifetime than men, supporting our hypothesis. Even among current users, women were two times more likely to be using fairness products than men. Although there have been no published studies comparing men and women’s use of fairness products in India, these findings support other work in the broader field of body image (4, 7), which indicates that societal expectations are much higher with regard to women’s appearance than men’s. It is likely that the general trend for women to have higher levels of body dissatisfaction than men, and a greater likelihood of being judged on appearance (7), plays a role in these gender differences. Furthermore, the messages related to increased cultural capital as an outcome of fairer skin also appear to target women more than men, especially in advertisements as described by Li et al. (4), which may be the reason for greater use among women. There are also many more products available for, and branded for, women than men. These results suggest that female consumers should be prioritized in further research and intervention efforts addressing the use of skin fairness products in India.

Among users, men were significantly younger than the women in this study. It is possible that increased availability of fairness products for men in recent years has contributed to greater use among younger male consumers, along with advertisements with male movie actors promoting the use of these products. The age of initiation was not significantly different across women and men. Thus, it is also possible that women continue using these products into middle age, while men may discontinue use after a certain age. Identifying age groups most likely to use skin fairness products for extended periods is worthy of further study as the long-term effects of fairness product use have not been sufficiently studied and it may provide useful information for populations to be targeted in public health interventions.

Within the current users, a considerable minority (17%) reported adverse side effects. This is a cause for public health concern in a market where there is no regulation of the products or the ingredients in the products. Furthermore, even when a single use of a product may be deemed “safe,” the regular use of some skin fairness products can lead to an accumulation of chemicals in the liver and kidneys which can cause damage to these organs (27). Women were significantly more likely to report side effects than men, which might be related to the earlier proposition that women might be using fairness products for longer periods of time. The type and duration of side effects as well as the social and economical consequences of these should be studied further. Furthermore, the fact that 17% of the users reported some kind of adverse reaction to using the products suggests that strong action needs to be taken at a policy level to regulate the ingredients and monitor the indiscriminate sale of these products. This supports past studies (12, 13) that have found that many products caused adverse side effects. Others have also reported side effects in other countries (14, 15), which has led to the banning of certain products in African countries.

Almost half (44.6%) of the users reported that the media influenced them in some way to start using fairness products. However, participants more strongly endorsed the subscale on family and peer ideas about fairness being desirable than any of the other subscales. It is possible that family members and peers are influenced by media as well, leading them to put pressure on participants to use fairness products, which would be the indirect impact of media. Interestingly, in terms of gender differences in reasons for using fairness products, men were significantly more likely to endorse items related to the connection between fairness and attractiveness as well as perceived pressure from family members and peers than women. This finding is all the more interesting given that more women reported using fairness products both currently and at some point in their life. This is contrary to our hypothesis that women would be more likely to relate attractiveness and cultural capital with fairness than men. As there is a dearth of research on gender differences as pertains to skin fairness product use, it is difficult to identify reasons for this variation and further research with samples of women and men is required.

Most women non-users endorsed reasons related to the side effects and efficacy of fairness products for non-use, while men were more likely to not use them for socioeconomic reasons. Given that more women are users and have reported side effects within the sample of users, it is possible that products marketed and sold to women are more potent and contain more harmful ingredients. In addition, as described earlier, women might use fairness products for a longer period of time, which might increase their susceptibility to side effects. As the men in the study were younger, it is possible that they may have less disposable income available for purchase of fairness products. However, the internal consistency of the socioeconomic reasons subscale was suboptimal; therefore further research with improved measurement is needed to replicate this result and to explore reasons for these findings.

While the results of this study point to areas for future research, there are some limitations to the study that should be acknowledged. As a result of lack of validated scales in this area, the assessment of attitudes toward use or non-use of fairness products was done using a scale that has not been previously validated. However, in this study, the reliability for the new measure appeared to be good. The sample while large, was conveniently obtained with a requirement of literacy and thus, may not be considered representative of the population of Mumbai or India. The scales used to assess beliefs about use of fairness products were different from the measure created to assess beliefs about not using them. Thus, it was not possible to directly compare the two groups on their beliefs. Furthermore, the scale created to assess beliefs among non-users and sub-optimal reliability and factor structure and, thus, results obtained with that scale must be interpreted with caution.

However, there were some key strengths of the study, primary of which is the fact that this is the first quantitative study examining beliefs regarding fairness products in an Indian city. Data were collected from a large sample in community settings. Although there were more women, there were a substantial number of men among both the users and non-users. In addition, the findings here support previous qualitative research (3, 4). No other studies have examined gender differences in the use of fairness products as well as gender differences in beliefs about the use or lack of use of fairness products.

There are crucial public health implications of the findings of this study. In terms of research, there are important avenues for future research. Since men chose not to use fairness products for socioeconomic reasons, future studies can include an assessment of the socioeconomic impact of use of fairness products among those who are regular users. It would be useful for future studies to quantitatively examine the physical and psychological impact of long-term use of fairness products along with exploring the relationship with other body image and related issues (low self esteem, high body dissatisfaction, lack of self confidence, perception of cultural capital). The findings on the media prompting people to start using fairness products suggests the need for further research on the impact of advertisements of these products.

From the point of view of public health practice, there are several potential areas of intervention. Primary among these is the design of programs to reduce stigma and prejudice associated with skin color and its connection with people’s judgment of appearance, beauty, attractiveness, and social status. This can be done *via* several media platforms and by education programs developed with a purpose to create more awareness and acceptance of the natural diversity of skin color that exists in India. It is essential that public health education efforts address differences between men and women in their beliefs about fairness and incorporate this in the design of their interventions. Also evident is the need to create more awareness about the adverse side effects of using fairness products. This can be done *via* consumer awareness programs at the state, national, and local levels. Most importantly, there is an obvious need for regulation of fairness products by the government. The regulation should take place at the level of ingredients in the product as well as at the level of advertising and marketing of products.

In conclusion, the results of this study shed light on the use of fairness products and beliefs about fairness in a metropolitan sample of women and men in India, one of the biggest global markets for skin fairness products. Peers, family, and media evidently play a role in influencing decisions to use fairness products and the desire to be more fair, beautiful, and attractive were the most frequent reasons for using these products. It is also notable that most individuals who were not current users reported not using products due to socioeconomic or lack of efficacy reasons. Fewer respondents endorsed a belief that they did not want to be fairer. Thus, the notion of enhanced cultural capital associated with fairness appears to be a strongly held belief even among those who are not current users of fairness products. These results support quantitative and qualitative research conducted in Asian countries and among Indians elsewhere that has reported a widely held belief of the connection between attractiveness, cultural capital, and fairer skin (23, 28). This is an important area worthy of further study, as similar studies in the field of body image have suggested that people report less self-confidence if they are dissatisfied with their appearance and this affects several aspects of their daily life and functioning (29). Furthermore, due to the adverse health consequences and potential to reinforce racism and health disparities, skin fairness product use warrants further research, advocacy, and intervention among public health professionals.

## Ethics Statement

In accordance with ethical guidelines governing research in India, institutional approval needs to be obtained for any research, which was done in this study. In addition, it is not necessary to obtain approval for the study from an ethical review board, especially as the study did not involve data collection with vulnerable participants and had no funding source. Nevertheless, all the procedures followed in the study complied with the guidelines laid down by ethics bodies in India. The participants were informed that the study was an exploratory investigation of the use of fairness products in India and that they would be asked to complete a questionnaire on their reasons for use or lack of use of fairness products. Participants were given information on the purpose of the study. They were informed that there would be no compensation for taking part in the study and that there were no known risks to participating. They were informed that participation was voluntary. No identifying information was collected from the participants, as such the questionnaires were anonymous and the risk of data protection issues was minimized. Once verbal consent was obtained, participants were provided with a paper version of the questionnaire and were asked to complete it in the presence of a research assistant. The questionnaires were administered in English. One large educational institution provided the researchers with permission to collect data in their classrooms. Data from the community sample (other than the educational institution) were collected from apartment complexes where the researchers were provided with permission to collect data. Certain companies also provided permission to collect data from their employees.

## Author Contributions

HS researched the previous literature, conceptualized the study, designed the questionnaire, did preliminary analysis, and wrote portions of the manuscript. PD researched and updated the background literature, provided critical inputs to bring focus to the manuscript, and wrote portions of the manuscript, including the introduction and results. NC analyzed the data and provided input on the other sections. All authors approve the final version of the manuscript, ensure the accuracy and integrity of the work, and agree to be accountable for all aspects of the work.

## Conflict of Interest Statement

The authors declare that the research was conducted in the absence of any commercial or financial relationships that could be construed as a potential conflict of interest.
